# Symptomatic sinus bradycardia: A rare adverse effect of intravenous ondansetron

**DOI:** 10.4103/1658-354X.76492

**Published:** 2011

**Authors:** Md Shahnawaz Moazzam, Farah Nasreen, Shahjahan Bano, Syed Hussain Amir

**Affiliations:** *Department of Cardiac Anaesthesia, Frontier Lifeline & Dr K M Cherian Heart Foundation, Mogappair, Chennai, India*; 1 *Department of Anaesthesiology & Critical Care, J N Medical College, AMU, Aligarh, India*

**Keywords:** *Adverse effect*, *ondansetron*, *sinus bradycardia*

## Abstract

Ondansetron is a serotonin receptor antagonist which has been used frequently to reduce the incidence of post-operative nausea and vomiting in laparoscopic surgery. It has become very popular drug for the prevention of post-operative nausea and vomiting due to its superiority in-terms of efficacy as well as lack of side effects and drug interactions. Although cardiovascular adverse effects of this drug are rare, we found a case of symptomatic sinus bradycardia in a 43-year-old female patient, going for laparoscopic cholecystectomy, who developed the same after she was given intravenous ondansetron in operation theater during premedication. Hence, we report this case, as the rare possibility of encountering bradycardia effect after intravenous administration of ondansetron should be born in mind.

## INTRODUCTION

Ondansetron is a selective serotonin [5-hydroxytriptamine 3 (5-HT_3_)] receptor antagonist which has been found to be highly effective in reducing the incidence of post-operative nausea and vomiting (PONV). It blocks these receptors in the brainstem as well as in the gut wall. Ondansetron has become a very popular pre-medicant for the prevention of PONV due to its superiority in - terms of efficacy as well as lack of side effects and drug interactions. Although cardiovascular adverse effects are rare, there have been reports of atrial fibrillation, cardiac dysarrhythmia and fatal ventricular tachycardia following intravenous administration of ondansetron.[1–3] We report a case of rare adverse effect of intravenous ondansetron which presented as symptomatic sinus bradycardia as it might be helpful in drawing attention to this possible adverse effect.

## CASE REPORT

A 43 year-old female of physical status ASA--I presented for laparoscopic cholecystectomy in routine operation theater with chronic cholecystitis with cholelithiasis. She had no history of cardiovascular disease, was not taking any regular medication and had no known allergies. Prior to pre-medication, the heart rate was 86 beats per minute (bpm) with normal sinus rhythm on cardio-scope and blood pressure was 128/86 (100) mm Hg. After intravenous cannulation with 18-Gauge catheter, ringer’s lactated fluid was started and 4 mg of ondansetron was administered slowly in running intravenous line. After 2-3 minutes, the patient complained of the impending faint and giddiness. Subsequently, she became unconscious and had respiratory arrest. Pulse oximeter showed SpO_2_ to be 98% and started decreasing rapidly. Cardioscope showed sinus bradycardia with a rate of 24 bpm and blood pressure was 102/72 (82) mm Hg. Immediately, the patient was intervened with mask ventilation with 100% oxygen. Within 2 minutes SpO_2_ increased to 100%, but heart rate was 26 bpm. Then, 0.5 mg atropine was administered intravenously, no response was seen within 3 minutes, then 0.5 mg atropine was repeated intravenously. After second dose of atropine was given, heart rate increased and reached 124 bpm with blood pressure 138/90 (106) mmHg. She regained spontaneous respiration and consciousness. She became well oriented within the next 5-6 minutes. Operation was postponed on that day. On the next day, trans-thoracic echocardiography was done and showed normal anatomical, physiological and mechanical functions. After 3 days, she was operated for the same without any complication.

## DISCUSSION

Ondansetron blocks emetogenic impulse both at their peripheral origin and their central relay. It blocks the depolarizing action of serotonin through 5-HT_3_ receptors on vagal afferents in the gut as well as nucleus tractus solitarius (NTS) and chemoreceptor trigger zone (CTZ). Ondansetron is considered safe and very effective for the prevention and treatment of PONV. Hence, it became the first choice anti-emetic drug for use during the perioperative period. However, there have been reports highlighting its possible cardiovascular adverse effects. It has reportedly caused atrial fibrillation within 15 minutes of intravenous injection,[1] bigeminy with ST segment depression, and sinus bradycardia followed by junctional rhythm with ventricular escape beats.[2] It is also reported to have induced fatal ventricular tachycardia.[3] Recently two cases have been reported to have severe bradycardia with respiratory arrest and loss of consciousness,[4] like the present case.

The sub-micro molecular affinity of ondansetron to “human ether a-go-go related gene (HERG)” encoded K^+^ channel underlies the prolongation of cardiac repolarization reported for this drug. Other serotonin antagonists like granisetron, dolasetron, tropisetron and palonasetron act on the Na^+^ and K^+^ channels to prolong QRS or QT interval resulting in ventricular arrhythmias.

Animal studies have proven that the 5-HT_3_ receptors present on vagal afferent nerve endings in the coronary bed evolve bradycardia, hypotension and apnea via the Von Bezold Jarisch (VBJ) reflex. Being a 5-HT_3_ antagonist, ondansetron attenuates these reflexes. Although several studies strongly reported the role of 5-HT_3_ antagonist in preventing the VBJ reflex, in the present case, the patient developed severe sinus bradycardia, apnea and moderate decrease in blood pressure (18%) after 4 mg intravenous administration of ondansetron. However, the mechanism of this paradoxical bradycardia is not clear. It is probably due to the fact that cardiovascular effects of 5-HT receptors are complex and consist of bradycardia or tachycardia, hypotension or hypertension and vasoconstriction or vasodilatation. Thus, in any given patient, blockade of 5-HT_3_ receptors by ondansetron will produce effects depending upon the pre-existing serotonergic activity in the parasympathetic and sympathetic limbs of the autonomic nervous system.[2]

## CONCLUSION

The mechanism of bradycardia in this case is not clear. A rare possibility of encountering bradycardia effect after intravenous administration of ondansetron should be born in mind. We suggest the judicious use of this drug under cardioscope monitor.
